# The microRNA-183/96/182 Cluster is Essential for Stereociliary Bundle Formation and Function of Cochlear Sensory Hair Cells

**DOI:** 10.1038/s41598-018-36894-z

**Published:** 2018-12-21

**Authors:** Ruishuang Geng, David N Furness, Chithra K Muraleedharan, Jinsheng Zhang, Alain Dabdoub, Vincent Lin, Shunbin Xu

**Affiliations:** 1 Department of Ophthalmology, Visual and Anatomical Sciences, School of Medicine, Wayne State University, Detroit, Michigan USA; 2 Department of Otolaryngology, School of Medicine, Wayne State University, Detroit, Michigan USA; 3 School of Life Sciences, Keele University, Keele, Staffs ST5 5BG United Kingdom; 4 Department of Communication Sciences and Disorders, College of Liberal Arts and Sciences, Wayne State University, Detroit, Michigan USA; 5 Biological Science, Sunnybrook Research Institute, Toronto, Ontario Canada

## Abstract

The microRNA (miR)-183/96/182 cluster plays important roles in the development and functions of sensory organs, including the inner ear. Point-mutations in the seed sequence of miR-96 result in non-syndromic hearing loss in both mice and humans. However, the lack of a functionally null mutant has hampered the evaluation of the cluster’s physiological functions. Here we have characterized a loss-of-function mutant mouse model (miR-183C^GT/GT^), in which the miR-183/96/182 cluster gene is inactivated by a gene-trap (GT) construct. The homozygous mutant mice show profound congenital hearing loss with severe defects in cochlear hair cell (HC) maturation, alignment, hair bundle formation and the checkboard-like pattern of the cochlear sensory epithelia. The stereociliary bundles retain an immature appearance throughout the cochlea at postnatal day (P) 3 and degenerate soon after. The organ of Corti of mutant newborn mice has no functional mechanoelectrical transduction. Several predicted target genes of the miR-183/96/182 cluster that are known to play important roles in HC development and function, including *Clic5*, *Rdx*, *Ezr*, *Rac1*, *Myo1c*, *Pvrl*3 and *Sox2*, are upregulated in the cochlea. These results suggest that the miR-183/96/182 cluster is essential for stereociliary bundle formation, morphogenesis and function of the cochlear HCs.

## Introduction

MicroRNAs (miRNAs) are small, non-coding RNAs of ~22 nucleotides in length that regulate gene expression by breakdown and/or translation inhibition of the messenger RNAs (mRNAs) of their downstream target genes^1–4^. miRNAs play important roles in almost all developmental and biological processes investigated thus far^1–4^. In the developing and mature mouse inner ear, hundreds of miRNAs have been found to be expressed in the cochlear and vestibular sensory epithelia^5–8^. They are involved in normal development and homeostasis of the inner ear, as well as in inflammatory processes and pathogenesis during noise-induced and age-related hearing loss^9–12^. Although conditional deletion of the miRNA biogenesis enzyme, dicer, demonstrated a vital role of miRNAs in inner ear development^7,8,13,14^, the functions and underlying molecular pathways of individual miRNAs in the inner ear and their roles in hearing are still poorly understood.

The miR-183/96/182 cluster was originally identified as a sensory organ-specific miRNA cluster^5,7,15–17^. In the inner ear, expression of this cluster is broadly distributed in the otic vesicle as early as embryonic day (E) 9. In later stages of inner ear development, it is confined to the sensory hair cells (HCs) and spiral ganglion cells^5,7,18^. Its expression is highly dynamic with tightly-controlled spatial and temporal patterns along different turns of the cochlea from embryonic to adult mouse^7^, and a radial and longitudinal gradient during chick ear development^19^. Studies in zebrafish suggest that miR-96 is required for hair cell formation^20^, while double knock-down of miR-183 and miR-182 also produces an inhibitory effect on hair cell formation^20^, suggesting that miR-183 and miR-182 are necessary for the normal function of the inner ear. Point mutations in the seed sequence of miR-96 lead to progressive non-syndromic hearing loss in mice and humans^21–24^. In spite of the high sequence homology between miR-96 and miR-182/miR-183, the latter two are unable to compensate for the functional defects caused by the mutations in the seed sequences of miR-96. The phenotypes caused by these point mutations of miR-96 are considered a result of both loss- and gain-of-function effects^21–24^, because they not only cause loss of regulation of normal target genes of wild-type miR-96, but also create new downstream target genes of the mutant miR-96. Further examination has shown that one of the point mutations causes arrest of HC development and maturation^23^. These data suggest that miR-96 plays a predominant role among members of the miR-183/96/182 cluster in inner ear function. It is uncertain whether miR-182 and miR-183 also play a significant role in mammalian inner ear development and function.

To understand the physiological functions of the miR-183/96/182 cluster, we generated a knockout (KO) mouse model using a gene trap (GT) embryonic stem cell clone, miR-183C^GT/GT^, in which the miR-183/96/182 cluster gene was inactivated by a GT construct^17^. Previously, we showed that inactivation of the cluster results in syndromic retinal dystrophy with multisensory defects including a circling phenotype, typical of vestibular defect^17^. This result suggests that loss of function of the miR-183/96/182 cluster leads to significant functional defects of the inner ear. Here, we characterize the impact of inactivation of the miR-183/96/182 cluster on inner-ear development and hearing functions. Our results demonstrate that loss of function of the miR-183/96/182 cluster causes severe defects in HC differentiation, morphogenesis and function, leading to profound congenital syndromic deafness in mice.

## Results

### miR-183/96/182 cluster KO mice have severe balance defects and profound deafness

**Figure 1:**
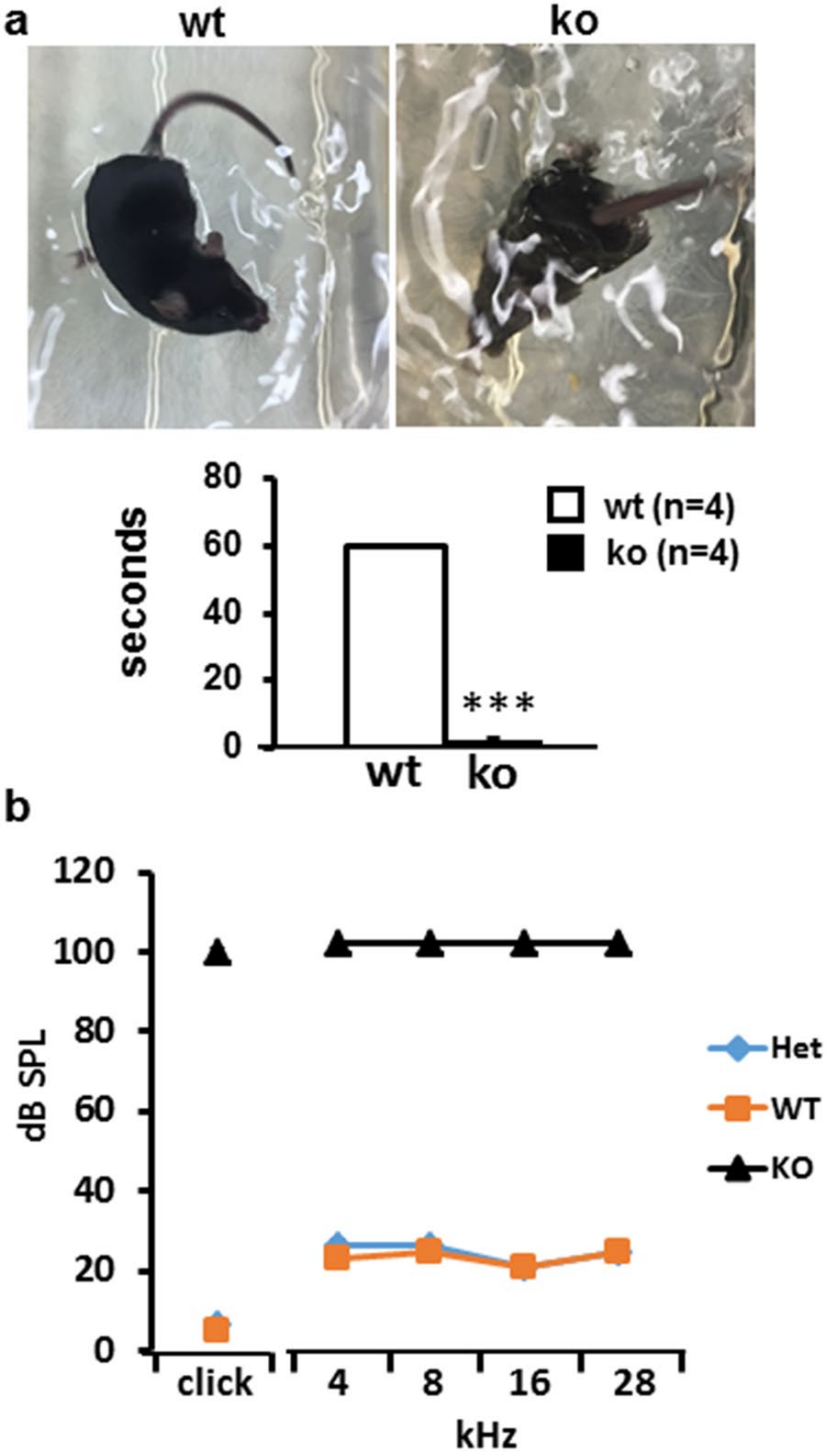
Balance and hearing dysfunction in miR-183/96/182 cluster KO mice. (**a**) Swimming test in 3 week-old WT and KO mice showing that a KO mouse was unable to keep its head above water. (**b**) Hearing test shows ABR threshold of WT (n = 3), heterozygote (n = 3) and KO mice (n = 5) at 3 weeks old.

Previously, we showed that the miR-183/96/182 KO mice have a circling behavior as early as the first week after birth^17^. To further test the defects in balancing, we performed a swimming test on young adult (3 weeks old) KO mice, and their wild-type (WT) littermates. The result revealed that, while the WT control mice swam well, kept calm and never lost balance during the test, the KO mice lost their balance and were unable to keep their heads above the water within seconds after being put in the water bath, confirming a vestibular defect (Fig. 1a).

To test whether inactivation of the cluster affects hearing, we performed auditory-brain stem response (ABR) test in 3-week-old mice. The results showed that the KO mice had no response to acoustic stimuli, whereas the heterozygotes and WT controls similarly showed normal hearing thresholds (Fig. 1b). These data suggest that inactivation of the miR-183/96/182 cluster in the cochlea resulted in profound hearing loss.

### The miR-183/96/182 cluster KO mice show severe defects in HC morphogenesis

**Figure 2:**
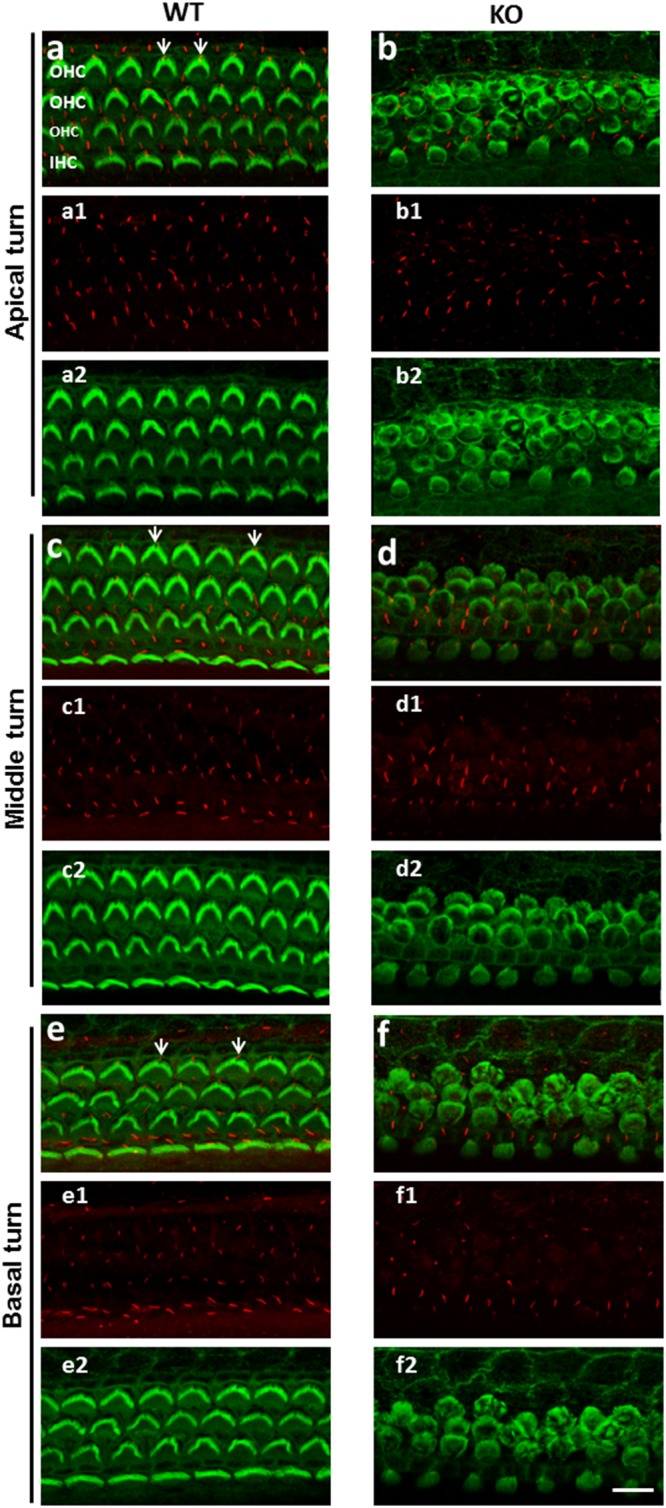
Confocal images of phalloidin staining and co-immunofluorescence of γ-tubulin of the apical surface of the cochlear sensory epithelia of P3 WT and KO mice. Phalloidin staining labels the F-actin (green) and anti-γ-tubulin (red) detects cilium structures. The precise arrangement of three rows of outer hair cells (OHCs) and one row of inner hair cells (IHCs) separated by alternating spaced supporting cells (SCs) in apical (**a**-a2), middle (**c**-c2) and basal turn (**e**-e2) is visible in the cochlea of WT mice. a1, c1 and e1 are images of γ-tubulin staining only, while a2, c2, e2 are single-channel images of phalloidin staining. γ-tubulin staining reveals a single kinocilium located on the mediolateral side of each HC at the vertex of the V-shaped stereocilia bundle (arrows in **a**,**c**,**e**) and there is also a cilium visible on each supporting cell apex. KO mice have abnormal HC spacing and alignment, their apices appear circular (**b**-b2, **d**-d2, **f**-f2); most mutant HCs lack kinocilia, although cilia similar to that in WT are visible on SCs. The scale bar = 10 μm.

At postnatal day (P) 3, the organ of Corti, and in particular the HCs, of the KO mice showed several abnormalities compared with WT controls. Phalloidin staining for actin filaments revealed the normal checkboard-like pattern of the HCs and supporting cells (SCs) in the WT reticular lamina, whilst tubulin-labelled ciliary extrusions were identifiable on both HCs, where they were adjacent to the bundle (known as the kinocilium), and SCs (Fig. 2a,c,e). However, in the KO mice, hair cell apices were abnormal, appearing circular rather than heart-shaped; and the hair bundle was malformed. Most HCs of the KO mice lack kinocilia, although the ciliary protrusion was retained on the SCs (Fig. 2b,d,f). These abnormalities were detected along the entire length of the cochlea, as indicated by comparable images from apical, middle and basal regions (Fig. 2b,d,f).

**Figure 3:**
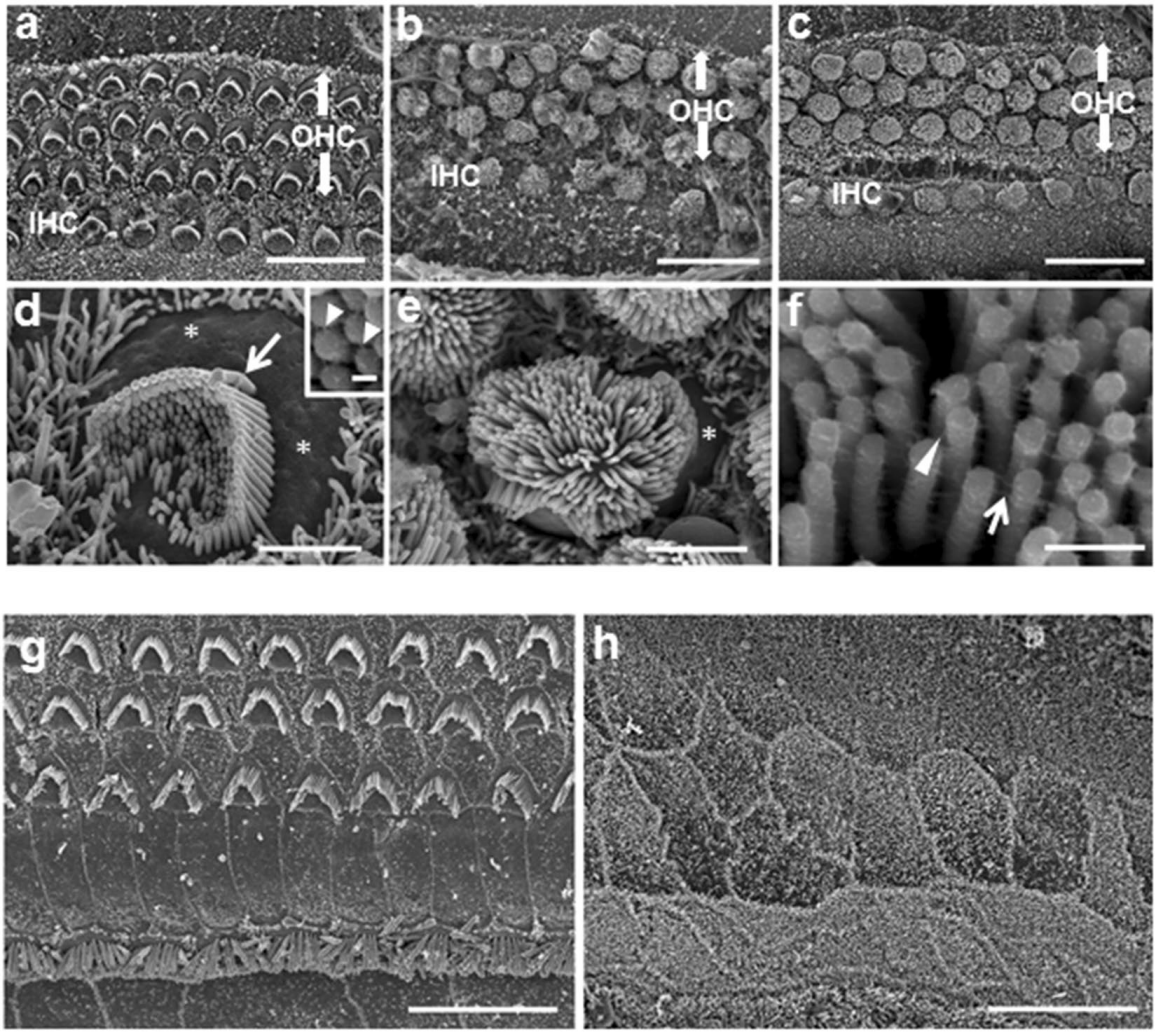
Scanning electron microscopy (SEM) of organ of Corti in WT and miR-183/96/182 cluster KO mice. **a**–**f**: P3 cochlea of WT (**a**,**d**) and KO (**b**,**c**,**e**,**f**) mice. **g,h**: P18 WT (**g**) and KO (**h**) mice. At P3, at low magnification (**a**–**c**), HCs in the WT mice are organized into the normal three rows of OHCs and one row of IHCs (**a**). The apical region of the cochlea is shown here. In the KO mice, the HCs appear dis-organized (b, apical turn; c, basal turn), with apical turn more severely affected (**b**). At higher magnification (**d**–**f**), the apical surface of P3 WT mice shows the normal staircase pattern of the stereocilia bundle, with additional shorter stereocilia, stereocilia-free zone (*) and kinocilium (arrow), typical of this age (**d**). Inset in (**d**): detailed view of ranked stereocilia in the WT with tip-link-like filaments connecting the shorter and taller stereocilia (arrowheads). In the KO mice, the staircase is undifferentiated; the stereocilia are all of a similar height and the bundle occupies virtually the whole apical surface with a reduced stereocilia-free zone at the periphery (* in **e**). Closer inspection of the stereocilia reveals links near the tips (arrowhead) and sides (arrow) of the bundle, but no distinct tip links are visible (**f**). By P18, the stereocilia are absent in KO mice (**h**) and the HCs replaced by a non-specific cell type in contrast to mature hair bundles in WT mice (**g**). Scale bars: (**a**,**c**,**g**,**h** = 15 μm, **b** = 12 μm, **d**,**e** = 2 μm,**f** = 300 nm).

**Figure 4:**
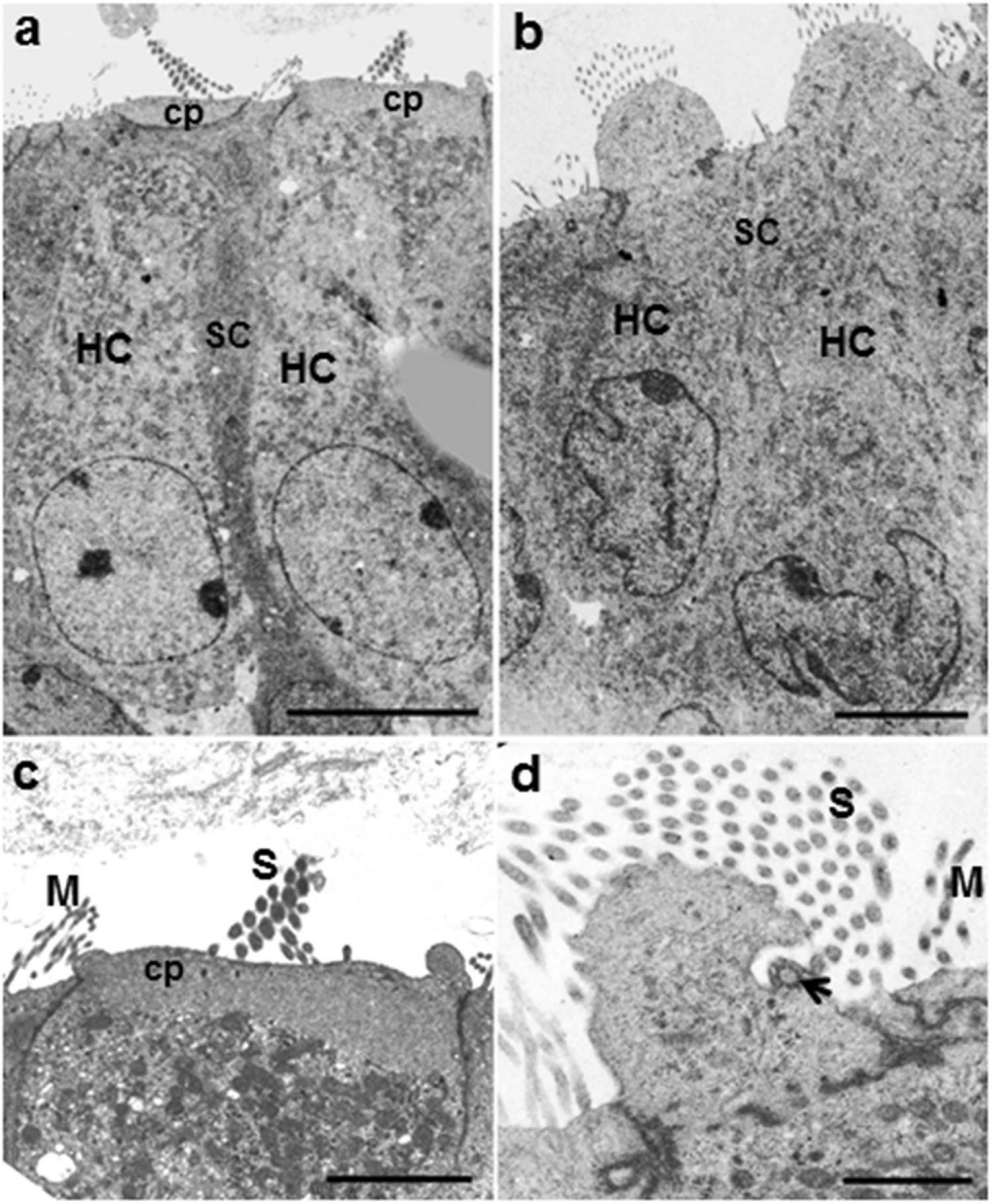
Transmission electron microscopy of organ of Corti sections in P3 WT (**a**,**c**) and the miR-183/96/182 cluster KO mice (**b**,**d**). In WT mice (**a**), outer hair cells (HC) are separated by phalangeal projections of Deiter’s cells (SC); their cytoplasm is lighter than that of surrounding SCs, and nuclei (N) are elliptical. The apices of the HCs are flat. In the KO (**b**), the HC cytoplasm is of equal density to SCs; their nuclei (N) are irregular; the apices protrude upwards; and the SCs are poorly defined and irregular. The hair bundles in the WT (**c**) consist of a staircase of stereocilia (S) extending from the cuticular plate (Cp); the hair bundle stereocilia are thicker than microvilli (M) on the adjacent SCs, whereas in the KO (**d**), the stereocilia (S) are as thin as adjacent SC microvilli (M), and extend from the bulbous protruding apex of the cell where there is a kinocilium basal body remnant (arrowhead). Scale bars A, B = 5 um; C, D = 2 um.

These abnormalities were further confirmed by electron microscopy (EM) (Figs 3 and 4). In the KO mice, the rows of HCs and SCs were disorganized to a small degree (Fig. 3b,c), with the apical region (Fig. 3b) more affected; the circular apices of the HCs were protruded (Fig. 3b,c compared to Fig. 3a; Fig. 4b compared to Fig. 4a). The hair bundles consisted of thin microvilli-like stereocilia of approximately equal length, reminiscent of immature stereociliary bundles from earlier embryonic stages (Figs 3e and 4d), in contrast to the control, where the staircase pattern of stereocilia was formed, the stereocilia thicker than adjacent microvilli on SCs (Figs 3d and 4c). The peripheral, stereocilia-free zone of the apical surface of HCs, which is visible in the WT (Fig. 3d), was substantially narrower, with the hair bundle covering virtually the entire apical surface in the KO mice (Fig. 3e). These abnormalities were evident in both apical and basal regions (Fig. 3b,c respectively).

At higher magnification, the stereocilia could be seen to be linked by fine filaments in the KO mice (Fig. 3f), but the lack of staircase organization meant that tip links could not be identified. The kinocilium basal body was observed by transmission electron microscopy (TEM), even where a complete kinocilium was absent (Fig. 4d).

The TEM observations also showed that whereas the cytoplasm of the HC bodies was apparently lighter than in the SCs in WT (Fig. 4a), the density of HC cytoplasm was comparable to that of SCs in the KO mice. It was difficult to distinguish the cytoplasm of HCs from the SCs (Fig. 4b). The HC nuclei of the KO mice were distorted (Fig. 4b), when compared with the WT control (Fig. 4a). The SCs between the HCs also showed evidence of distortion in the KO cochlea (Fig. 4a,b). Furthermore, the apical filamentous cuticular plate, clearly visible in the WT (Fig. 4a,c), was not detectable in the KO (Fig. 4b,d).

By P18, scanning electron microscopy (SEM) showed that the HC bundles were completely lost, and HC apices could not be detected in the KO mice (Fig. 3h). The epithelium was composed of non-specified cell types, perhaps expanded SCs (Fig. 3h).

Additional phalloidin staining in P6 cochleae showed that, although most HCs in the KO mice still existed, they appeared, qualitatively, to be more disorganized and have some cell loss (Supplemental Fig. 1), suggesting that HC degeneration starts between P3 and P6.

### Mechanoelectrical transduction (MET) is non-functional in the miR-183/96/182 KO mice

**Figure 5:**
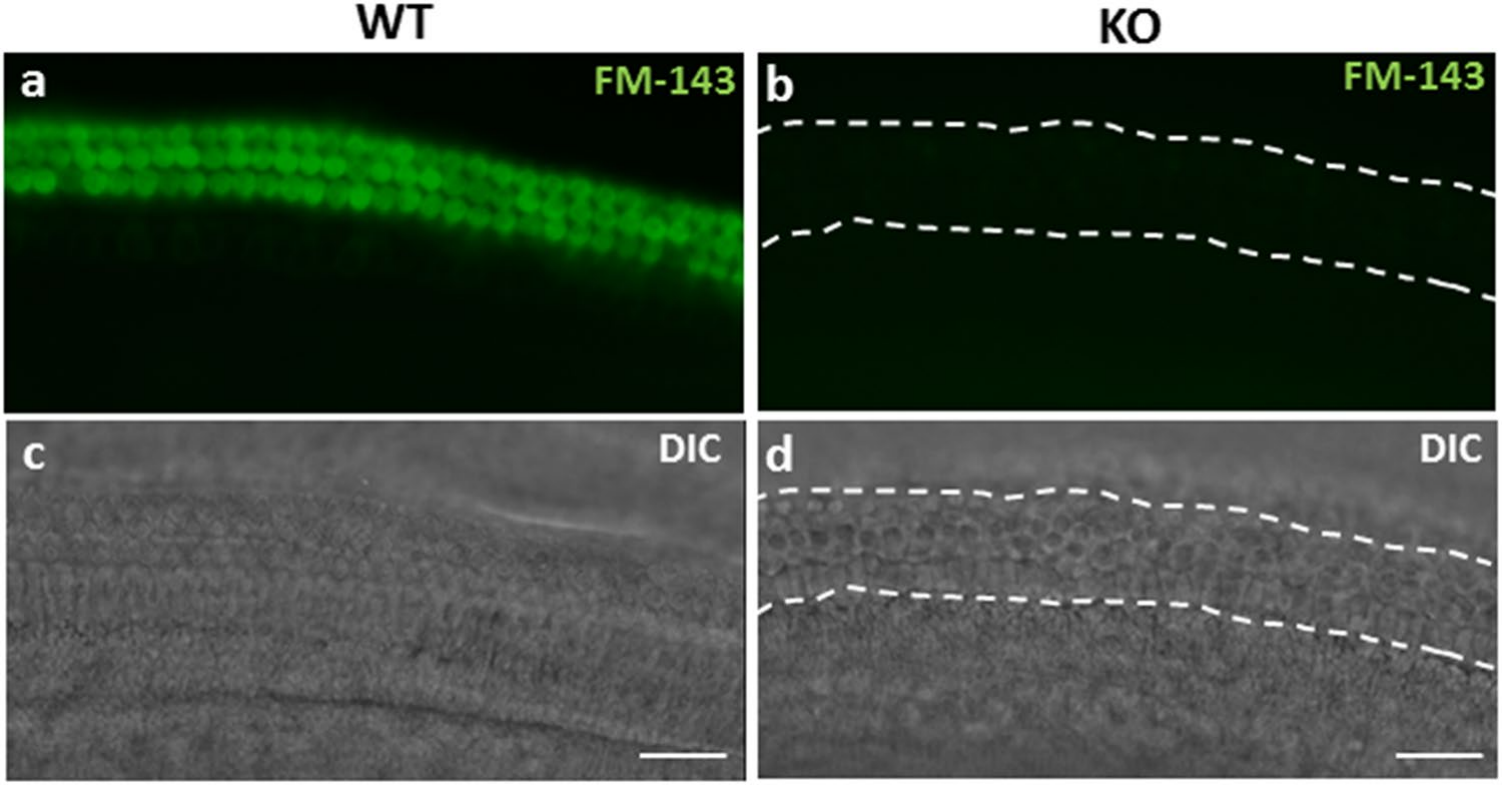
FM1–43 dye uptake in P1 WT (**a**,**c**) and KO mice (**b**,**d**). Panel **a** shows dye uptake in the middle turn of WT cochlear OHCs. There was also uptake in the IHCs in WT; however, it is invisible in this picture because IHCs were in different focus plane. No uptake in KO cochlear hair cells (**b**). Panels c and d are DIC images of same regions of HC epithelium as shown in a and b, respectively. Dotted lines in b & d delineate the medial (lower) and later (upper) boundaries of the organ of Corti. The scale bars = 20 μm.

Normally, the tip links in HC bundles operate ion channels to initiate MET during deflections of the HC bundle^25–28^. The lack of a staircase pattern and tip-links in the KO mice suggest that their HCs are unable to perform MET. To test this hypothesis, we performed a FM1–43 dye uptake assay. Our result showed that, FM1–43, a fluorescent dye that enters through functional MET channels^29^, failed to load in the HCs of cochlear organotypic cultures derived from P1 KO mice (Fig. 5b), whilst dye uptake was evident as bright fluorescence in the HCs of WT organotypic cultures (Fig. 5a). Thus, as predicted, the mutant HCs lack functional MET.

### Stereociliogenesis related genes were upregulated in the mutant cochlear epithelium

**Figure 6:**
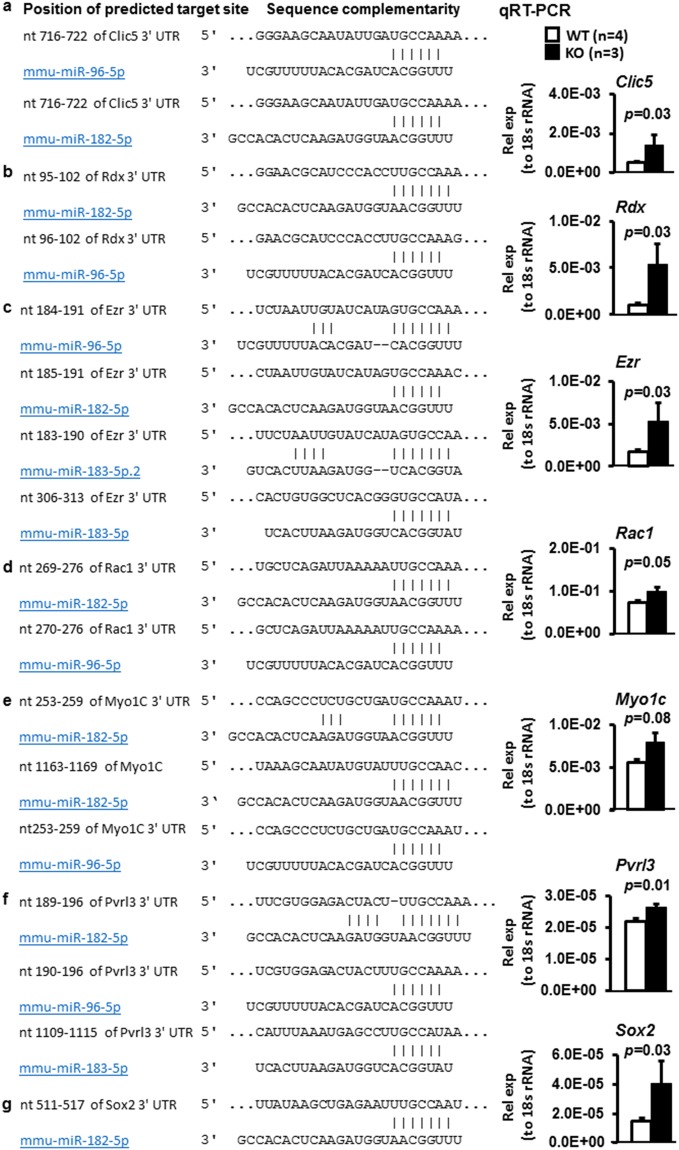
Sequence alignment and qRT-PCR analysis of predicted target genes of the miR-183/96/182 cluster in P1 cochlear epithelia. Rel exp (to 18 s rRNA): relative expression level normalized to 18 s rRNA as an endogenous loading control. n = 4 for WT; n = 3 for KO.

Several proteins including *Clic5*, ezrin (*Ezr*), radixin (*Rdx*), taperin, and myosin VI have been shown to act together in organizing stereociliary actin filaments and their interactions with the plasma membrane^30–32^. *Clic5*, *Rdx* and *Ezr* are predicted target genes of miR-183/96/182 cluster (Fig. 6a–c)^33,34^. To test whether these genes are regulated by miR-183/96/182 cluster in the inner ear *in vivo*, we isolated total RNA from cochlear sensory epithelia of P1 mice. Quantitative (q)RT-PCR assays showed that the expression of *Clic5* (2.69 fold), *Rdx* (5.17 fold) and *Ezr* (2.99 fold) was significantly upregulated in the cochlea of KO mice vs. WT littermate controls, suggesting that miR-183/96/182 cluster regulates these genes *in vivo*; and loss of miR-183/96/182 cluster resulted in dysregulation of these genes (Fig. 6a–c).

### The expression of other predicted target genes known to play important roles in HC development and function was significantly up-regulated in KO mice

In addition to the above-mentioned protein complexes, *Rac1*^35,36^, *Pvrl3*^37,38^, *Myo1c*^39,40^ and *Sox2*^41–44^, which are known to play important roles in inner ear HC development and function, are also predicted targets of the miR-183/96/182 cluster (Fig. 6d–g). And therefore, we included these genes in our qRT-PCR analysis. Our result showed that the expression of these genes was significantly upregulated [*Rac1* (1.33 fold), *Pvrl3* (1.21 fold), *Myo1c* (1.43 fold), *Sox2* (2.71 fold)] in the P1 cochlear epithelia of KO mice (Fig. 6d–g), suggesting that they are likely targeted by the miR-183/96/182 cluster in the cochlear sensory epithelia *in vivo*.

**Figure 7:**
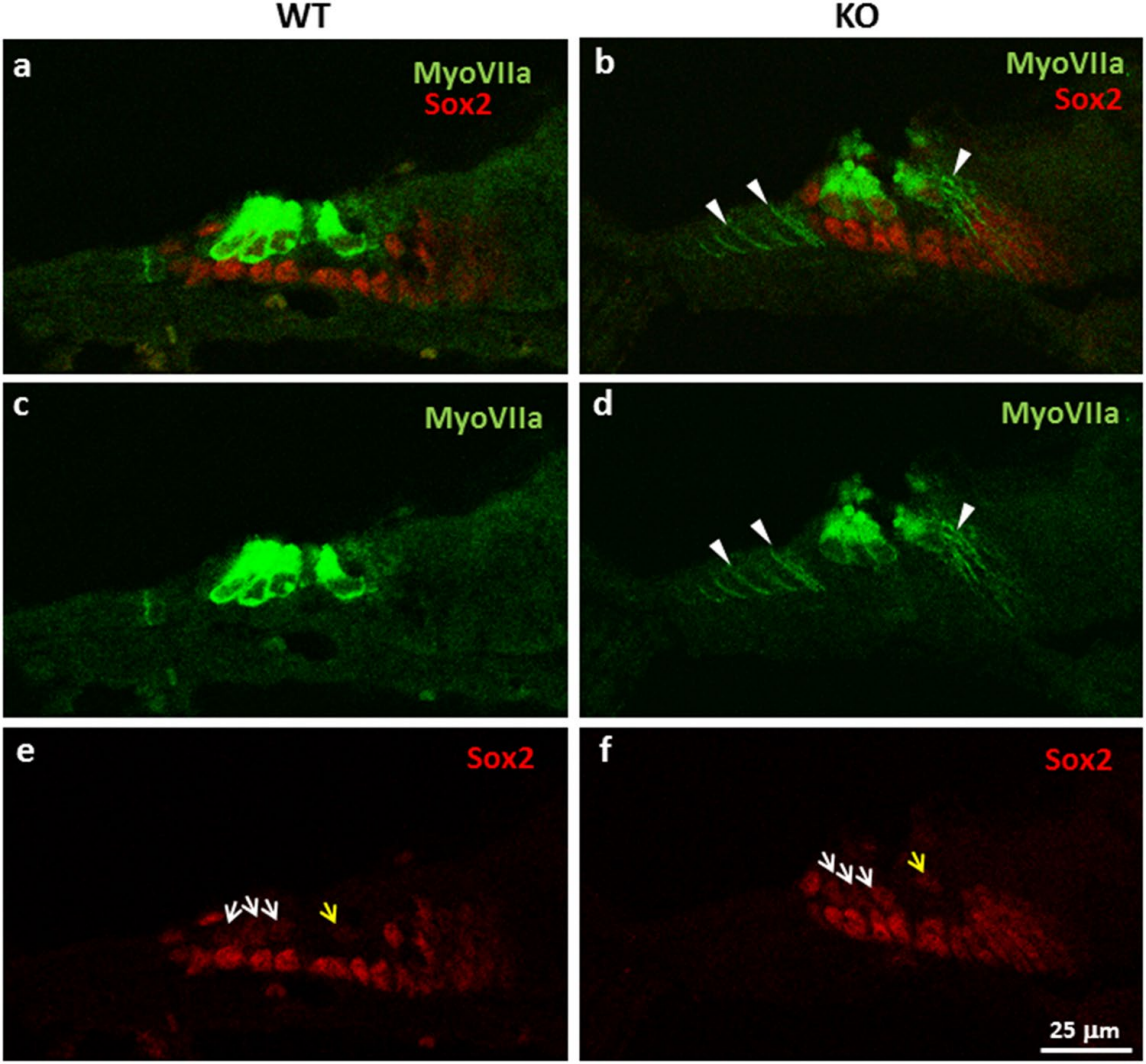
Immunostaining of cochlear sensory epithelium with anti-*Sox2* (red) and *Myo7a* (green) antibodies in P1 WT (**a**,**c**,**e**) and KO mice (**b**,**d**,**f**). White arrows in e and f show *Sox2* staining in the nuclei of OHCs; yellow arrows show that in the nuclei of IHCs. White arrowheads in b and d point to the “ectopic” staining of *Myo7a* in a subgroup of cells in the GER and LER regions. Similar results were observed in the cochleae from 3 KO and 2 WT mice.

*Sox2* plays an essential role in the formation of the prosensory domain and HC differentiation during inner ear development via transient expression in HCs before P2 but retained expression in SCs subsequently^41–45^. To examine the cellular localization and protein expression level of *Sox2*, we performed co-immunofluorescence of *Sox2* and a HC-specific marker, *Myo7a*, in P1 cochlear sensory epithelia. Similar results were observed in the cochleae of 3 KO and 2 WT mice. Representative qualitative images are illustrated in Fig. 7. Our result showed that *Sox2* staining in the nuclei of HCs appeared qualitatively more intense in the KO (Fig. 7f) vs. WT mice, especially in IHC (Fig. 7e), but there is no apparent qualitative difference in the SCs. This is consistent with increased expression of *Sox2* in the cochlear epithelia of KO mice at mRNA level by qRT-PCR analysis (Fig. 6g) and suggests that the miR-183/96/182 cluster targets *Sox2* in HCs *in vivo*. The staining of *Myo7a* appeared qualitatively much weaker in KO HCs, compared to WT controls, indicating impaired HC differentiation or maturation in KO mice. Intriguingly, in contrast to WT controls, the SCs surrounding the HCs in the greater and lesser epithelial ridges (GER and LER) appeared to be positive to *Myo7a* staining (arrowheads in Fig. 7b,d).

## Discussion

miRNAs are quantitative regulators of gene expression. One miRNA often targets multiple protein-coding genes in the same pathway or functional network, and imposes modest regulation on each of them. This contributes to the maintenance of the homeostasis of the pathway/network under physiological conditions. Defects in a miRNA can thus result in simultaneous dysregulation of multiple genes, leading to significant functional consequences when the composite impact passes a threshold^17,46–50^. Here we provide evidence that the miR-183/96/182 cluster provides a global regulation of the differentiation, morphogenesis and functional maturation of HCs and organization of the organ of Corti.

Our data show that, although HCs are generated, loss of function of the miR-183/96/182 cluster results in a multitude of defects in their development and function: (i) HCs are misaligned within the cochlear epithelium; (ii) the cuticular plate is not formed; the HC apex is protruded; and the stereociliary bundle fails to develop to maturity; (iii) there is loss of MET function; and (iv) by P18, the HCs have completely degenerated; the KO mice have a profound hearing loss. These data confirm that the miR-183/96/182 cluster plays important roles in the differentiation, morphogenesis and function of inner ear HCs, and extend previous findings of Weston *et al*.^7,51^ and Fan *et al*.^52^ by providing more structural and expression data relating to the effects of loss of the cluster specifically in the inner ear.

A point mutation in the seed sequence of miR-96 has been reported to cause defects in the maturation of stereociliary bundles in the *Dmdo/Dmdo* mice^22^. In these mice, the stereocilia form a partially differentiated bundle^22,23^, but development is arrested around P0^23^. These defects are considered to be a result of loss of function of the wild-type miR-96, as well as the gain-of-function effect of the mutant miR-96, which creates abnormal target genes not normally targeted by the wild-type miR-96^22,23^. In spite of their high sequence homology to miR-96, miR-183 and miR-182 fail to compensate/rescue the effects caused by the point mutation of miR-96. Therefore, it is uncertain whether miR-183 and miR-182 have a functional significant role in HC development and differentiation in mammalian cochlea. Comparing to the *Dmdo/Dmdo* mice, the hair bundle defects in our miR-183/96/182 cluster KO mice are apparently more severe, as the apical surface of HCs retains a more immature appearance with an unranked cluster of microvilli-like stereocilia, the apical surface is protruded above the HC cell body, and the overall organization of the organ of Corti is also affected. These data suggest that complete loss of function of the miR-183/96/182 cluster resulted in more severe defects in inner ear HC development and differentiation than the point mutation of miR-96 alone, and that miR-183 and miR-182 may also play a significant role in HC development and functional differentiation. This is consistent with the observation in zebrafish that simultaneous knockdown of two or three members of the cluster results in an additive inhibitory effect on HC formation^20^. Creation and characterization of miR-182/miR-183 double KO and miR-96 single KO mice are needed to further reveal their physiological functions in inner ear HC development and functions.

As we were preparing this manuscript, Fan *et al*. reported an independent conventional knockout mouse of the miR-183/96/182 cluster by homologous recombination^52^. Similar to what we described here in the miR-183C^GT/GT^ KO mice both temporally and morphologically, the conventional KO mice also showed severe defects in the stereociliary bundle development and HC maturation, misalignment of HCs and profound hearing loss^52^. These striking similarities between our miR-183C^GT/GT^ KO and the independently-created conventional miR-183/96/182 cluster KO mice further strengthen the conclusion that miR-183/96/182 cluster plays an essential role in HC development and function. This also confirms that the miR-183C^GT/GT^ model is a functional KO of the miR-183/96/182 cluster, arguing against the suggestion that the miR-183C^GT^ allele could be hypomorphic^52^. This is further supported by our initial characterization of the expression of miR-183/96/182 cluster in the sensory organs of the miR-183C^GT/GT^ mice^17^. We demonstrated that, resembling the complete inactivation of this cluster in the retina, expression of miR-183, −96 and −182 in WT cochlea was at least 30-, 25- and 21-fold higher than in the cochlea of miR-183C^GT/GT^ mice by qRT-PCR analysis^17^.

Coupled with the morphological studies, our target prediction and gene expression analysis suggest that the miR-183/96/182 cluster imposes a global regulation on the differentiation and function of the organ of Corti through simultaneous regulation on multiple genes involved in various aspects of the development of HCs. Hair cells have complex cytoskeletal networks, especially in the apical part of the cell. The ezrin-radixin-moesin (ERM) family plays important roles in the morphogenesis of the actin cytoskeleton in various cell types^53–55^, including HCs^31,56^. In HCs, they interact with *Clic5* to form a complex at the ankle of stereocilia that may stabilize the linkage between plasma membrane and actin cytoskeleton^30^. In the miR-183/96/182 cluster KO mice, upregulation of the ERM genes, *Rdx* and *Ezr*, as well as *Clic5* in the cochlear epithelia may have interrupted the balance of gene expression and the development of the apical part of HCs. This perhaps contributes to the failure of cuticular plate formation, the apical surface protruding from the cell body and the stereocilia retaining immature phenotype. Supporting this notion, Fan *et al*. reported that the conventional KO mice had increased and disorganized acetylated microtubules below the apical surface of the hair cells where the cuticular plate would otherwise form^52^.

*Rac1* is a member of the Rho family small GTPases, and has been shown to regulate planar cell polarity (PCP) and morphogenesis of the stereociliary bundle of HCs^35^. Deletion of *Rac1* in the otic epithelium resulted in a reduced number of, and severely shortened HCs, with defects in PCP and morphogenesis of the bundle^35^. Our data suggest that loss of the miR-183/96/182 cluster results in dysregulation of *Rac1* in P1 cochlear sensory epithelia, which may contribute to the phenotypes in the stereociliary development in the KO mice.

*Pvrl1* and *3*, also known as *Nectin-1* and *−3*, are immunoglobin-like adhesion membrane molecules. *Pvrl1* is expressed in HCs, while *Pvrl3* is expressed in SCs. This mutually exclusive expression pattern is involved in the HC-SC interaction and the development of the checkerboard-like cellular pattern of the cochlear sensory epithelium^37,38^. Increased expression of *Pvrl3* in the cochlea epithelia of KO mice suggest that miR-183/96/182 cluster is likely to regulate *Pvrl3* in HCs *in vivo* and affect HC-SC interaction and cellular organization in the cochlear epithelium.

A major aspect of HC function is mechanoelectrical transduction. Our data showed that, in the miR-183/96/182 KO mice, MET no longer functions. The absence of a mature staircase of ranked stereocilia means that the tip-link complex that underlies transduction^57^ may not be formed; and indeed, specific tip links have not been identifiable here. *Myo1c* is one of several unconventional myosins that have been implicated in the slow form of adaptation in HC MET by regulating tip-link tension^58–64^. *Myo1c* upregulation in auditory epithelia of the miR-183/96/182 cluster KO mice suggests a possible role of these miRNAs in regulating tip-link function and adaptation.

*Sox2* has a dual role in the specification of sensory competence and HC differentiation^43,44^. Its early expression is required to formation of the prosensory domain and HCs^41–43^. However, its subsequent downregulation in HCs must occur to allow HCs to become fully differentiated^43,44^. In mice, after P2, its expression is undetectable in HCs but retained in SCs^41,45^. Increased expression of *Sox2* in the HCs of P1 KO mice suggests that loss of miR-183/96/182 cluster contributes a delayed downregulation of *Sox2* in HCs, and consequently, may affect the functional differentiation of HCs.

We anticipate that many other genes are also regulated by the miR-183/96/182 cluster and contribute to the overall phenotype in HC development and function. A genome wide analysis of gene expression changes in HCs of this mutant model would provide further information and deeper understanding of the roles of miR-183/96/182 cluster in HC development and functions. Phenocopying the mutant phenotype by overexpressing some of these target genes would help confirm their roles in mediating the functions of the miR-183/96/182 cluster in HCs.

The observation of apparent ectopic expression of *Myo7a* in cells in the GER and LER surrounding the HCs is intriguing. Increasing evidence has shown that a subset of SCs in these regions may act as cochlear progenitor cells with a capacity of regeneration and differentiation into HCs in neonatal mice, especially after damage^65–68^. In our P1 KO mice, the HCs are morphologically and functionally defective. It is reasonable to hypothesize that, through unknown mechanisms, defective HCs may have signaled and induced progenitor cells to generate more HCs; the ectopic expression of *Myo7a* has reflected this attempt. However, extensive follow-up studies will be needed to test this hypothesis.

In conclusion, we have demonstrated major effects of the miR-183/96/182 cluster in the development of the organ of Corti and also HCs. Further characterization of HC development and function in mutant mice with single or double knockout of members of the cluster^69,70^ is therefore warranted.

## Materials and Methods

### Mice

Animal care and husbandry were handled in accordance with the National Institute of Health (NIH) guidelines. All protocols were approved by the Institutional Animal Care and Use Committee, Wayne State University. The miR-183/96/182 cluster KO mice, miR-183C^GT/GT^, are on a 129S2/BL6-mixed background and were originally derived from a gene-trap embryonic stem cell (ESC) clone as described previously^17,71^. Neonatal (P1~6) and 3 weeks old mice KO mice, their heterozygous (miR-183C^GT/+^) and WT littermates (miR-183C^+/+^) were used in this study. At least three mice per genotype were used in each experiment. The age and number of mice used for each experiment are further specified in the method description below and in the text and figure legends.

### Swimming test

Young adult (3 weeks old) KO mice and their WT littermates were gently placed in a tank filled with warm tap water and the swimming behavior recorded by a camera of iPhone8 (Apple). They were rescued from the water within 15 seconds (s) after they lost their balance or were unable to keep their heads above the water. The duration from the time when the mice were put in water to when they lost their balance was recorded. All WT mice kept calm and never lost their balance even after 1 minute in water; accordingly, the duration for all WT mice was arbitrarily recorded as 1 minute for quantification purpose.

### ABR recording

ABR recording was performed as previously described^72^. Briefly, 3 weeks old mice were anesthetized with ketamine (100 mg/kg) and xylazine (20 mg/kg) and placed in a soundproof chamber (Tracoustical, Inc., Austin, Texas) during testing. Their body temperature was maintained by placing them on a homeothermic heating pad. ABR testing was carried out using a TDT system (Tucker-Davis Technologies, Alachua, Florida). Platinum subdermal needle electrodes (MFI Medical Equipment, Inc., San Diego, CA) were inserted at the vertex of the head, and ventrolateral to both ears. The mice were presented with 4–28KHz pure tone stimuli at an intensity starting at 100 decibel sound pressure level (dB SPL) and decrementing in 10-dB steps; this sequence was repeated in 5-dB steps until the lowest intensity that evoked a reproducible ABR waveform (peaks I–IV) was detected.

### Immunofluorescence

The inner ears of P1 pups were dissected out from the temporal bone. After removal of the stapes, a small hole was made to open the apex; the cochlea was fixed by perfusion through the oval window and the hole at the apex with 4% paraformaldehyde (PFA) in 1X PBS solutions at 4 °C, and then left in the fixative overnight. After 3 rinses with 1XPBS, the cochlear sensory epithelium was dissected out. For immunostaining of kinocilia, the tissue was soaked in blocking solution (5% normal donkey serum and 0.1% Triton X-100 in 1X PBS) at room temperature for one hour (h), then incubated with anti-acetylated γ-tubulin antibody (Sigma. 1:250) in blocking solution at 4 °C overnight. After three 15-minute (min) washes with PBS containing 0.1% Triton X-100, a Alexa Fluor-568-conjugated secondary antibody (Invitrogen) was applied together with Alexa Fluor-488 Conjugated phalloidin (Invitrogen), which stains actin-rich structure, stereocilia, for 1 h at room temperature. After 3 washes with 1X PBS, the tissue was mounted on slide and images were taken with a confocal microscope (Leica TCS SP8).

For immunofluorescence of cryosections, inner ears of P1 mice (3 KO and 2 WT) were dissected out and fixed with 4% PFA in 1X PBS by perfusion through the oval window and a hole opened at the apex and incubation for 1 h at room temperature on a shaker. After washing with 1X PBS for 15 mins, three times, the inner ears were incubated consecutively in 10%, 15%, 20%, 25% and 30% sucrose in 1X PBS for 30 mins at each concentration at room temperature and then kept in 30% sucrose in 1X PBS at 4 °C overnight. Then the inner ears were transferred to OCT and kept at 4 °C, overnight. After degassed with vacuum, the inner ear was frozen and cryosectioned at 12 μm thickness. After blocking, the cryosections were stained with anti-Myo7A (Proteus Biosciences Inc.. 1:500) and anti-Sox2 (Santa Cruz Biotechnology. 1:250) at room temperature for 2 h. After washing with 1XPBS, the sections were incubated with secondary antibodies (donkey anti-goat Alexa Fluor 546 and donkey anti-rabbit Alexa Fluor 488 secondary antibodies. Invitrogen. 1:1000) for 1 h at room temperature. The images were taken with a confocal microscope (Leica TCS SP8).

### Electron microscopy

Cochleae of P3 and P18 mice were excised from the temporal bone and fixed by perfusion as described above using 2.5% glutaraldehyde in 0.1 M sodium cacodylate buffer containing 2 mM calcium chloride and incubation for 2 h. They were stored in 1/10th fixative until further processing. For postfixation, the samples were washed in sodium cacodylate buffer and then immersed in 1% osmium tetroxide in sodium cacodylate for 1 h.

For SEM, cochleae were dissected and then prepared using the OTOTO technique^73^. Briefly, segments were incubated in alternating solutions of osmium tetroxide (3X, 2 h) and saturated thiocarbohydrazide (2X, 20 mins) with six washes in water between each change. They were subsequently dehydrated through an ethanol series and critical point dried using a Polaron drier, mounted on platinum stubs using adhesive carbon pads, and examined in a Hitachi S4500 field emission SEM at 5 kV.

For TEM, samples were fixed and dehydrated as for SEM, but then embedded in Spurr’s resin^73^, sectioned at 70–100 nm and the sections collected on copper grids. Grids were stained in uranyl acetate and lead citrate and examined in a JEOL 100 S TEM. Images were recorded on Acros Neopan 35 mm negatives and digitized using a HP Canonscan 9000 negative scanner.

### Cochlear organ culture and FM1–43 labeling

The function of the MET channels of mouse cochlea was assessed by FM1–43 labeling as described previously^29^. Briefly, cochlear sensory epithelia of P1 mice were carefully dissected out in cold Hepes-buffered Hanks’ balanced salt solution (HBHBSS. Sigma) and cultured in DMEM/F12 with 7% Fetal calf serum (FCS) (Fisher Scientific) and 5 mg/ml ampicillin (Sigma) on Cell-Tak-coated glass coverslips in Mat-Tk dishes (MatTek Corporation) at 37 °C. After overnight culture, the cochlea was rinsed briefly in HBHBSS and then dipped in HBHBSS containing 3 µM FM1–43 (ThermoFisher Scientific) for 10 seconds (s) and rinsed 3 times in fresh HBHBSS solution. The coverslip was immediately mounted on slide and imaged with fluorescent light microscope (Leica) equipped with epifluorescence optics and FITC filters (488 nm excitation, 520 nm emission).

### RNA isolation and qRT-PCR

Cochlear epithelia from P1 mice were dissected in HBHBSS, and quickly transferred to RNAlater (Ambion/ThermoFisher Scientific). Total RNA was prepared using RNeasy micro kit (Qiagen) following manufacturer’s instructions. On-column DNase I treatment was performed to avoid traces of DNA contamination. qRT-PCR assays of protein-coding genes were performed using QuantiFast SYBR Green RT-PCR kit and QuantiTect primers (Qiagen) with 18 s rRNA as an endogenous loading control as we described previously^17,74–76^. The relative expression level of a gene of interest was normalized to 18 s rRNA. Briefly, the cycle threshold (Ct) value of the gene of interest (Ct_goi_) was normalized to the Ct value of 18 s rRNA (Ct_18s rRNA_) and was calculated as DCt = Ct_goi_-Ct_18s rRNA_. Subsequently, the relative expression level of the gene of interest was calculated as 2^−DCt^.

### Statistical analysis

All data are shown as mean ± SEM. Two-tailed student *t* test was performed in the quantitative comparison in the swimming test. Mann-Whitney test (one-tailed) was employed to test the statistical significance between KO mice and WT controls in the qRT-PCR analysis using the GraphPad Prism 6 software (GraphPad Software, Inc., La Jolla, CA). Since this is a simple comparison between KO and WT controls, no multiple test correction was employed in the *p*-value calculation.

## Supplementary information

Supplementary info
