# Supplementary material for: The microRNA-183/96/182 Cluster is Essential for Stereociliary Bundle Formation and Function of Cochlear Sensory Hair Cells
Source: Sci Rep. 2018 Dec 21;8:18022. doi: 10.1038/s41598-018-36894-z (PMC6303392; doi:10.1038/s41598-018-36894-z)
Supplement: Supplementary file 1 — Supplementary info [file 41598_2018_36894_MOESM1_ESM.pdf]

Supplementary material

The microRNA-183/96/182 Cluster is Essential for Stereociliary Bundle Formation and Function of Cochlear Sensory Hair Cells

Ruishuang Geng[1,2*], David N Furness[3], Chithra K. Muraleedharan[1], Jinsheng Zhang[2,4], Alain Dabdoub[5], Vincent Lin[5], Shunbin Xu[1*]

1. Department of Ophthalmology, Visual and Anatomical Sciences;
2. Department of Otolaryngology, School of Medicine, Wayne State University, Detroit, Michigan, USA.
3. School of Life Sciences, Keele University, Keele, Staffs, ST5 5BG, United Kingdom
4. Department of Communication Sciences and Disorders, College of Liberal Arts and Sciences, Wayne State University, Detroit, Michigan, USA
5. Biological Science, Sunnybrook Research Institute, Toronto, Ontario, Canada

* Correspondence and requests for materials should be addressed to S.X. (sxu@med.wayne.edu) and R.G. (email: ruishuang@gmail.com).

Supplementary figure

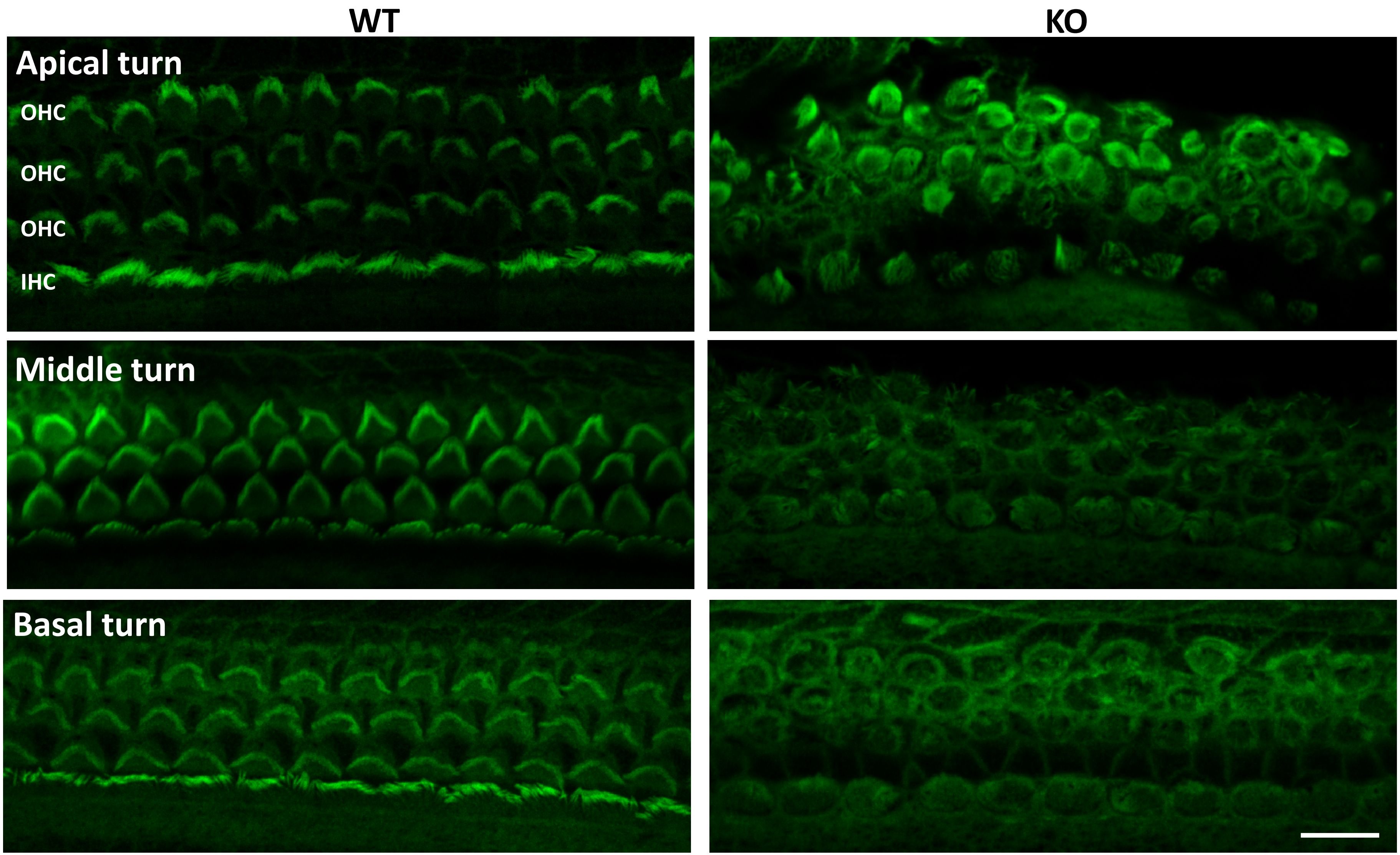

Figure S1. Confocal images of phalloidin staining of the apical surface of the cochlear sensory epithelia of P6 WT and KO mice. OHCs: outer hair cells; IHCs: inner hair cells. Scale bar=10 μm.
